# Transmicron: accurate prediction of insertion probabilities improves detection of cancer driver genes from transposon mutagenesis screens

**DOI:** 10.1093/nar/gkac1215

**Published:** 2023-01-09

**Authors:** Carl Bredthauer, Anja Fischer, Ata Jadid Ahari, Xueqi Cao, Julia Weber, Lena Rad, Roland Rad, Leonhard Wachutka, Julien Gagneur

**Affiliations:** TUM School of Computation, Information and Technology, Technical University of Munich, 81675 Munich, Germany; Institute of Molecular Oncology and Functional Genomics, TUM School of Medicine, Technical University of Munich, 81675 Munich, Germany; Center for Translational Cancer Research (TranslaTUM), TUM School of Medicine, Technical University of Munich, 81675 Munich, Germany; Computational Health Center, Helmholtz Zentrum Munich, Neuherberg, Germany; Institute of Molecular Oncology and Functional Genomics, TUM School of Medicine, Technical University of Munich, 81675 Munich, Germany; Center for Translational Cancer Research (TranslaTUM), TUM School of Medicine, Technical University of Munich, 81675 Munich, Germany; TUM School of Computation, Information and Technology, Technical University of Munich, 81675 Munich, Germany; TUM School of Computation, Information and Technology, Technical University of Munich, 81675 Munich, Germany; Graduate School of Quantitative Biosciences (QBM), Ludwig-Maximilians-Universität München, 81377 Munich, Germany; Institute of Molecular Oncology and Functional Genomics, TUM School of Medicine, Technical University of Munich, 81675 Munich, Germany; Center for Translational Cancer Research (TranslaTUM), TUM School of Medicine, Technical University of Munich, 81675 Munich, Germany; Center for Translational Cancer Research (TranslaTUM), TUM School of Medicine, Technical University of Munich, 81675 Munich, Germany; Institute for Experimental Cancer Therapy, TUM School of Medicine, Technical University of Munich, 81675 Munich, Germany; Institute of Molecular Oncology and Functional Genomics, TUM School of Medicine, Technical University of Munich, 81675 Munich, Germany; Center for Translational Cancer Research (TranslaTUM), TUM School of Medicine, Technical University of Munich, 81675 Munich, Germany; German Cancer Consortium (DKTK), 69120 Heidelberg, Germany; Department of Medicine II, Klinikum rechts der Isar, TUM School of Medicine, Technical University of Munich, 81675 Munich, Germany; TUM School of Computation, Information and Technology, Technical University of Munich, 81675 Munich, Germany; TUM School of Computation, Information and Technology, Technical University of Munich, 81675 Munich, Germany; Computational Health Center, Helmholtz Zentrum Munich, Neuherberg, Germany; Institute of Human Genetics, TUM School of Medicine, Technical University of Munich, 81675 Munich, Germany

## Abstract

Transposon screens are powerful *in vivo* assays used to identify loci driving carcinogenesis. These loci are identified as Common Insertion Sites (CISs), i.e. regions with more transposon insertions than expected by chance. However, the identification of CISs is affected by biases in the insertion behaviour of transposon systems. Here, we introduce Transmicron, a novel method that differs from previous methods by (i) modelling neutral insertion rates based on chromatin accessibility, transcriptional activity and sequence context and (ii) estimating oncogenic selection for each genomic region using Poisson regression to model insertion counts while controlling for neutral insertion rates. To assess the benefits of our approach, we generated a dataset applying two different transposon systems under comparable conditions. Benchmarking for enrichment of known cancer genes showed improved performance of Transmicron against state-of-the-art methods. Modelling neutral insertion rates allowed for better control of false positives and stronger agreement of the results between transposon systems. Moreover, using Poisson regression to consider intra-sample and inter-sample information proved beneficial in small and moderately-sized datasets. Transmicron is open-source and freely available. Overall, this study contributes to the understanding of transposon biology and introduces a novel approach to use this knowledge for discovering cancer driver genes.

## INTRODUCTION

Carcinogenesis is an evolutionary process that results from the interplay of somatic mutations and selective pressure (1–3). Discovering mutational cancer driver genes, i.e. genes affected by mutations that are under positive selection in a given cancer type is an important task in the pursuit to understand cancer aetiology and identify potential therapeutic targets.

Transposon-based forward genetic screens based on insertional mutagenesis have contributed to this task by identifying large sets of driver genes in mouse models (4,5). PiggyBac (PB) and Sleeping Beauty (SB) are two commonly used transposon systems to identify driver genes in both solid and hematopoietic malignancies (6–10). DNA transposons are genetic elements that can change their genomic position through a cut-and-paste mechanism (11–13). They are two-component systems, consisting of a transposable element (transposon) and a mobilizing enzyme (transposase). The transposase facilitates the excision of the transposon from a donor locus as well as its reintegration at another genomic position. Reintegration of transposons is highly specific for TA (SB) or TTAA (PB) motifs, respectively (14).

Depending on the adjustable transposon cargo sequence, the reintegration can cause the activation or inactivation of nearby genes. For instance, gene activation can be caused if the transposon contains an internal promoter. Conversely, transposons can also function as bidirectional loss-of-function gene traps by inserting internal splice acceptors and bidirectional poly(A) sites (15,16). Hence, transposon insertions can drive carcinogenesis by targeting both proto-oncogenes and tumour suppressor genes. Transposon mutagenesis has been found to be advantageous over classical retroviral mutagenesis due to its short-acting effects on targeted genes (17), its applicability to any cell type in the body (16), and its ability to discover functionally relevant elements outside of mutated genes (10).

In forward mutagenesis screens, tumour cells are enriched for insertions conferring selective advantages: Cancer develops when oncogenic insertions ultimately cause uncontrolled proliferation. Consequently, tumour tissue will contain many clones of the cell carrying the oncogenic insertions that induced carcinogenesis. Therefore, cancer genes can be determined from transposon screens by identifying common insertion sites (CISs), i.e. regions with more insertions than expected by chance across multiple independent samples. In order to distinguish genuinely positively selected (driver) insertions from non-oncogenic (passenger) insertions that clonally expanded along with driver insertions in a particular tumour, evidence from multiple tumours is required (18).

There are two major classes of statistical methods for detecting CISs in transposon-based screens. On the one hand, so-called locus-centric methods consider all possible regions in the genome with enrichment for insertions, independently of genome annotation. This includes methods based on counting insertions in windows of fixed sizes (6,19–22) or on Gaussian kernel convolutions (CIMPL: 18,23). Null distributions and control for multiple testing have been obtained analytically using the Poisson distribution (PRIM: 21, TAPDANCE: 22), empirically through Monte Carlo simulations (19,20,20) or random permutations (18). Locus-centric methods have the advantage of being unbiased of prior knowledge and therefore potentially identifying any genomic region enriched for insertions, possibly at various levels of resolution. However, this flexibility comes at the price of cumbersome post-processing interpretation methods to attribute the detected CISs to genes and a potentially high multiple testing burden. On the other hand, gene-centric methods focus on insertion counts within annotated genetic regions, typically extended to cover the promoter. Gene-based methods do not rely on arbitrarily determined window sizes (24,25), do not require cumbersome attribution of the detected CISs to genes (26,27), and may reduce the multiple testing burden, potentially increasing sensitivity. However, being limited to annotated elements (28), they are less flexible regarding the scale and the scope of the CISs to be analysed. Of note, despite the discrete nature of insertion count data, none of the existing gene-based approaches use count distributions. Instead, exiting gene-centric methods rely on chi-squared tests to compare either observed gene-wise insertion counts (gCIS: 24,28), or the number of independent tumours harbouring at least one insertion in a given gene (SB Driver: 27) to a corresponding expectation generated based on the number of potential target sites in each gene.

Importantly, for both locus-centric and gene-centric approaches, the identification of CISs is typically conducted under the assumption that all regions of the genome have an equal probability of harbouring insertions (29). In particular, current methods to analyse transposon screens assume that all TA sites (for SB) and TTAA sites (for PB) on the same chromosome have an equal likelihood of transposon insertions in the absence of selective pressure (30). However, it has been shown that transposon systems display biases in their insertion behaviour. For example, PB is known to be biased towards CpG islands, transcribed units, and markers of open chromatin (31–33). SB shows non-random integration behaviour on a micro-scale since it is strongly influenced by local DNA-deformability (34–36). On larger scales, many studies found SB insertions to be relatively unbiased (34–38). Others reported biases concerning transcribed regions, gene density, and epigenetic features (33,39–41). Notably, De Jong *et al.* (39) found biases for SB that are similar to the biases of PB in their comprehensive study on retroviral and transposon insertions under non-selective conditions. Berry *et al.* (41) reanalysed two SB datasets from different laboratories and cell types using the same method of analysis. They found conflicting associations of insertion sites to genomic and epigenomic features, suggesting that the insertion site preferences of SB depend on the cell type or the experimental conditions.

Crucially, it has been shown that not accounting for these biases can lead to a substantial number of false-positive CISs (29). For instance, in their study on lentiviral mutagenesis screens, Biffi *et al.* (42) estimate the number of false positives by assuming that true CISs should contain more insertions than adjacent genomic regions. They mark 3 out of 9 CISs as potentially arising from benign integration bias rather than oncogenic selection. Others have compared CISs versus control datasets generated under minimal selective pressure: Wu et al. (43) report a false positive rate of 47% in a murine leukaemia virus screen. Starr *et al.* (20) find 6 CIS in their control samples, compared to 79 CIS in their tumour samples. Finally, De Jong *et al.* (39) report that 13% (SB) and 33% (PB) of CISs found in transposon mutagenesis screens overlap with integration hotspots from non-selective samples. However, none of the published tools to identify CISs from transposon screens takes the insertion site biases of transposon systems into account, although some authors acknowledge the problem (18,21).

**Figure 1.**
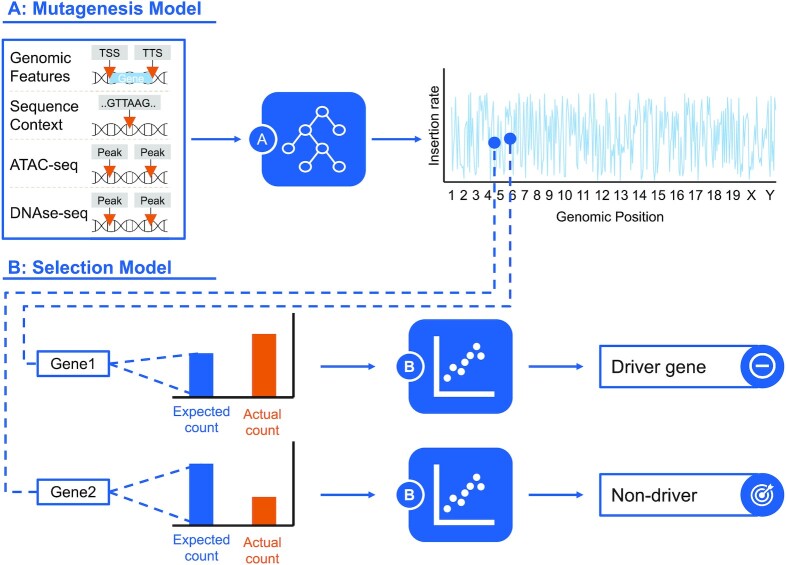
The Transmicron method. We learn to model neutral insertion rates by training a classifier to discriminate observed insertion sites from random control sites using various genomic and epigenomic features (**A**: mutagenesis model). Next, we estimate a selection coefficient for each genomic region by modelling observed insertion counts accounting for neutral insertion rates, sequencing depth, and chromosomal variations (**B**: selection model). Genomic regions with significantly increased selection coefficients are called. Genes from significantly enriched regions are predicted to be cancer driver genes. TSS: Transcription Start Site; TTS: Transcription Termination Site.

Here, we introduce Transmicron (Transposon Mutagenesis Screen Correction), a novel computational method to identify CIS controlling for transposon integration biases, which can be applied either in a locus-centric or in a gene-centric fashion. Our method comprises two steps (Figure 1). First, the insertion behaviour under a non-selective mutagenic process is modelled to generate an estimate of the background distribution of insertions (mutagenesis model). Second, this background distribution is compared to the actual, experimental distribution of insertions to identify genomic regions that harbour more insertions than expected (selection model). The selection model applies count-based Poisson statistics, as successfully implemented by PRIM and TAPDANCE (21,22), while simultaneously considering the distribution of insertions across samples, as introduced by SB Driver (27).

## MATERIALS AND METHODS

### Data generation

#### Generation of mouse strains and cohorts

Constitutive PB and SB transposase knock-in mice (Rosa26PB, Rosa26SB), Blmm3 mice and ITP transposon lines have been described earlier (9,44,45). In addition to the PB ITP mice analysed by Weber *et al.* (45), triple- and double transgenic SB mice were generated by crossing Rosa26^SB/+^;Blm^m3/m3^ with either ITP1-C;Blm^m3/m3^ or ITP2-M;Blm^m3/m3^. For this study, we analysed a cohort of diffuse large B cell lymphomas (DLBCLs) developed in PB ITP mice (described in Weber *et al.*, 45, *n* = 30) and SB ITP mice (*n* = 11) as well as tail tissue as a control tissue (*n* = 12 for PB and *n* = 11 for SB). All experimental mice were maintained on a mixed C57BL/6 × 129Sv × FVB background. Mice were kept in the animal facilities of the Wellcome Trust Sanger Institute, Hinxton/Cambridge, UK under specific-pathogen-free (SPF) conditions on a 12-h light/dark cycle, receiving food and water ad libitum. All animal experiments were carried out in compliance with the requirements of the European guidelines for the care and use of laboratory animals and were approved by the UK Home Office and the Institutional Animal Care and Use Committees (IACUC). Genotyping was performed according to Friedrich *et al.* (15).

#### Necropsy and immunohistopathological analysis

All animals were regularly monitored for signs of sickness. During necropsy, a gross inspection of all organs was carried out. Tumour tissue and the tail were sampled to isolate tumour and control DNA. Tissue samples were stored in RNAlater (Sigma) for subsequent DNA isolation. The histological procedure and immunohistochemical analysis to diagnose diffuse large B cell lymphomas were performed as described in Weber *et al.* (45).

#### DNA isolation

DNA was isolated using the Qiagen DNeasy Blood & Tissue Kit according to the manufacturer's instructions.

#### Quantitative transposon insertion site sequencing

QiSeq was performed as described in Friedrich *et al.* (15).

### Data preparation

We excluded insertions of the tail tissue samples from the data analysis unless stated otherwise. In addition to our newly generated DLBCL dataset, we analysed other datasets described in previous publications. Specifically, we examined screens from cohorts developing acute myeloid leukaemia (AML), cutaneous squamous cell carcinoma (cuSCC), hepatocellular carcinoma (HCC), and gastrointestinal tumours (Intestinal) (23,25,46), as well as insertions in mouse embryonic stem cells (mESC) (37). If insertion sites in these datasets had been mapped to the mm9 version of the mouse genome, a lift-over to mm10 was performed using the UCSC chain file. Some insertions were not precisely mapped to the canonical position within a TA / TTAA motif but shifted by a few nucleotides. We corrected for this shift assuming the misalignment to be an artifact. Moreover, insertions located on the chromosome harbouring the donor locus or on a sex chromosome were excluded in all cases. After filtering, 43,863 SB insertions (74%) and 158,358 PB insertions (88%) remained in our DLBCL datasets.

### Statistical model

#### Step 1: modelling mutagenesis

The mutagenesis model was used to estimate the probability of a transposon to insert at least once at genomic position. It was implemented as a random forest classifier trained to distinguish observed transposon insertion sites from randomly drawn TA / TTAA nucleotides from the entire genome (41). Thus, the classifier estimated the probability $\widehat y$ of a genomic position from the training set to be an observed insertion site (${y_m} = 1$) or a random control site (${y_m} =$0):

(1) $$\begin{equation*}{\widehat y_{m|T}} = P\left( {{y_m} = 1|m \in T} \right),\end{equation*}$$

where $T$ is the training set with observed transposon insertion sites and an equal number of random controls. The input features to the model were (i) sequence context (±10 bp) around locus $m$ as well as the distances of locus $m$ to (ii) the closest transcription start site (TSS), (iii) the closest transcription termination site (TTS), (iv) the closest gene body (valuing 0 for intragenic positions), (v) the closest DNAse-seq peak and (vi) the closest ATAC-seq peak. The positions of the genic features (features ii–iv) were retrieved from the UCSC RefSeq mm10 track. Unless stated otherwise, we used chromatin accessibility data (features v–vi) from previous studies (DNAse-seq: Geeven *et al.*, (47); GEO GSM1954931, ATAC-seq: Miller *et al.*, (48); GEO GSM2467459) of healthy mESC. Peaks were filtered for *q*-value <0.05 and a lift over to mm10 was performed (49). Sites overlapping with the positions of the features were encoded as 0. The mutagenesis model was implemented using scikit-learn (50) and trained by splitting the experimental data into a training set and a test set (90%/10%). Model parameters and hyperparameters were estimated based on 5-fold cross-validation within the training set and the model performance was assessed on the unseen test set. The hyperparameters ‘bootstrap’, ‘max_depth’, ‘max_features’, ‘min_samples_leaf’, ‘min_samples_split’, ‘n_estimators’ of the random forest classifier were optimized using a randomized search over 100 iterations. Subsequent fine-tuning of the hyperparameters yielded no significant performance improvements.

We modelled neutral transposon insertion counts ${k_m}$ using the Poisson distribution with a site-specific rate ${\lambda _m}$. To map the classifier prediction to the Poisson rate we first note that the probability of having one insertion at locus $m$ is:

(2) $$\begin{eqnarray*}P\left( {{y_m} = 1} \right) &=& p\left( {{k_m} \ge 1} \right) = 1 - p\left( {{k_m} = 0} \right) \nonumber \\ &=& 1 - exp\left( { - {\lambda _m}} \right).\end{eqnarray*}$$

The insertion probabilities on the single nucleotide level had to be aggregated across genomic intervals: It follows from the properties of the Poisson distribution, as well as Equations (1) and (2), that the insertion rate for the set of all insertion sites of the $j$th genomic region of interest (${\Lambda _j}$) is:

(3) $$\begin{eqnarray*}log\left( {{\Lambda _j}} \right) &=& log\sum\nolimits_{m \in j} {{\lambda _m}} \nonumber \\ &=& log \left( {\sum\nolimits_{m \in j} {log\left( {1 + {{\widehat \omega }_{m|T}} \cdot {f_0}} \right)} } \right),\end{eqnarray*}$$

where ${\widehat \omega _{ m |T}} = \frac{{{{\widehat y}_{ m |T}}}}{{1 - {{\widehat y}_{ m |T}}}}$ is the odds ratio of the classification probabilities, and ${f_0}$ is the ratio of negatives in the training set $T$ to the total number of TA/TTAA nucleotides in the genome.

#### Step2: modelling selection

On top of local insertion biases, which are modelled with the mutagenesis model, we control for two global biases that influence insertion counts in any particular experiments, namely chromosome biases (21) and sequencing depth. We estimate those two effects in a first step. To this end, we partitioned the genome into bins of 50 kb and counted the number of insertions per sample $i$ and genomic bin $j$. We next modelled the counts using a generalized linear model (GLM) with Poisson-distributed response variables where the expected counts ${E_{ij}}$ was:

(4) $$\begin{equation*}log\left( {{E_{ij}}} \right) = log\left( {{\sigma _i}} \right) + log\left( {{\chi _k}} \right) + log\left( {{\Lambda _j}} \right),\end{equation*}$$

where ${\sigma _i}$ models sequencing depth, ${\chi _k}$ models chromosomal effects, and ${\Lambda _j}$is a fixed parameter obtained from Equation (3).

Based on the estimates for ${\sigma _i}$ and ${\chi _k}$, we fitted a second GLM for every genomic region $j$ (e.g. a gene):

(5) $$\begin{equation*}log\left( {{E_{ij}}} \right) = {\beta _j} + log\left( {{\sigma _i}} \right) + log\left( {{\chi _k}} \right) + log\left( {{\Lambda _j}} \right),\end{equation*}$$

where ${E_{ij}}$ is the expected insertion count in genomic region $j$ in sample $i$ and ${\beta _i}$ was the selection coefficient of the genomic region $j$. We then tested whether ${\beta _j}$ is greater than zero (one-sided Wald-test). Transmicron can be run with $j$ being any type of user-defined genomic region. Unless otherwise stated, the results presented here were derived with UCSC RefSeq mm10 genes. The family-wise error rate (FWER) was controlled at the 5% level by correcting p-values using the Bonferroni method. We split the method into Equations (4) and (5) to allow parallel evaluation of (5).

### Comparing the performance of transmicron to existing methods

We benchmarked Transmicron against three established methods (CIMPL, PRIM, and SB Driver) (18,21,27). Following previous approaches (21,27), we relied on the cancer gene census (CGC) (51,52) as a catalogue of established cancer genes used for benchmarking, where not otherwise indicated. To test our approach for robustness to different catalogues of cancer genes, we also used the list of DLBCL genes published in Reddy *et al.* (53). As suggested by the authors in their method vignette (https://github.com/NKI-CCB/cimpl/blob/master/inst/doc/cimpl.pdf), CIMPL was implemented with the scale parameter ranging from 10 kb to 150 kb in 10-kb increments and the iterations parameter set to 10,000. For benchmarking, we used results obtained by setting the scale parameter to 10 kb, as this yielded the highest precision measured against the CGC. CIS were mapped to genes using CIMPL’s built-in mapping functionality. If multiple associated gene identifiers were found for a CIS, the gene was chosen at random.

Similarly, PRIM was implemented on window sizes from 10 kb to 150 kb in 10-kb increments. Because we lacked the required data, we ran the PRIM version without the PRI-correction factor that accounts for restriction site biases. As PRIM provides a built-in function to aggregate results over window sizes, the aggregated list of CIS was used for benchmarking. PRIM-CIS were mapped to genes by overlapping insertion sites with gene annotations from the UCSC RefSeq mm10 track. If a CIS overlapped with multiple genes, the assigned gene was chosen at random. Moreover, we enabled PRIM to run on PB datasets by supplying the number of TTAA nucleotides instead of TA nucleotides.

As suggested by the authors, SB Driver was implemented with the promoter cut-off set to 0 kb. No read depth filtering was applied. We excluded genes that are not hit in at least a certain number of tumours (3 tumours or 5% of all tumours, whichever is larger) (27). To ensure the comparability of the results, we consistently applied this threshold to all methods. Moreover, we selected Bonferroni multiple testing correction at 5% significance level for all methods.

As mentioned above, all results from running Transmicron presented here are based on running the method on the UCSC RefSeq mm10 annotation unless stated otherwise. To investigate to what extent our results are influenced by gene-based implementation we also ran Transmicron on 10 kb genomic windows and compared the results to CIMPL (scale parameter set to 10,000) and PRIM (window size set to 10 kb).

## RESULTS

### Transmicron successfully models selection

Transmicron comprises two conceptual steps: the mutagenesis model and the selection model. To delineate the contribution of each step, we start by assessing the added value of the selection model before systematically exploring the effects of integrating our mutagenesis model into Transmicron.

**Figure 2.**
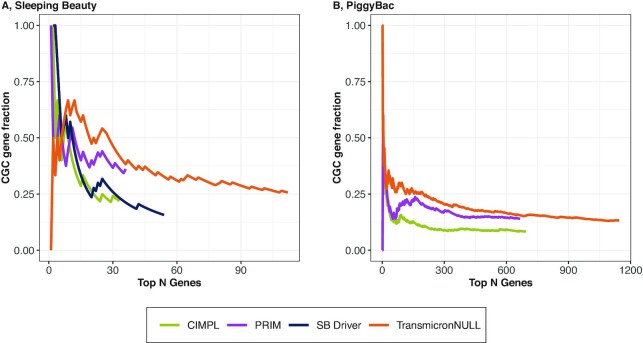
Proportion of known cancer genes for TransmicronNULL and existing tools. The fraction of driver genes indexed in the CGC database among sets of top N genes detected by each method for a Sleeping Beauty (11 tumours) (**A**) and a PiggyBac (30 tumours) (**B**) screen is shown. Results from TransmicronNULL, PRIM, and SB Driver Analysis were sorted by gene-wise p-value. If for any method, a gene was detected multiple times, the lowest p-value was used. Results for CIMPL were sorted by its peak height metric because this yielded higher precision than sorting by p-values. If two or more genes had identical p-values, the genes were assigned the same rank using the highest value. Curves stop at various ranks because only significant results (FWER < 0.05) are shown.

To analyse the selection model, we implemented a simplified version of Transmicron controlling only for the number of TA / TTAA sites per region (TransmicronNULL). Specifically, we set the insertion rate per region ${\Lambda _j}$ (Equations (4) and (5)) to the number of TA / TTAA sites in region $j$. We benchmarked TransmicronNULL in the gene-centric mode for which target regions are genes (Materials and Methods), against three available state-of-the-art methods (CIMPL, PRIM, SB Driver) by comparing the CGC gene fraction (proportion of cancer genes indexed in the Cancer Genes Census among all genes detected by each method) based on our DLBCL datasets (Figure 2A and B).

Reassuringly, all methods were enriched for known cancer genes in both DLBCL datasets. Moreover, we found TransmicronNULL to detect a considerably higher number of genes (FWER < 0.05) than existing methods in both the SB screen and the PB screen while demonstrating a higher CGC gene fraction across the top-ranked genes (Figure 2A and B). Specifically, in the SB dataset (11 tumours), TransmicronNULL detects more than twice as many significant genes (109 vs. 36) as the best performing competitor (PRIM), albeit with a slightly lower fraction of CGC genes (26% versus 36%, Figure 2A). Similarly, in the PB dataset (30 tumours), TransmicronNULL detected the highest number of significant genes (1,084) with a fraction of CGC genes slightly lower than that of PRIM (13% versus 14%). Moreover, the fraction of CGC genes was consistently higher for TransmicronNULL than for the other methods, with exceptions among the very top genes where results were noisy.

### PiggyBac and sleeping beauty display different biases in their insertion behaviour

Having demonstrated that our selection model offers a competitive approach to detect selected genes, we turned to explore the effects of controlling for known transposon insertion site biases (mutagenesis model). As a first step, we analysed our DLBCL datasets with regard to several genomic and epigenomic features previously shown to affect transposon insertion likelihood. In accordance with previous research (18,38,39,41), we analysed our DLBCL datasets with respect to three categories of features.

**Figure 3.**
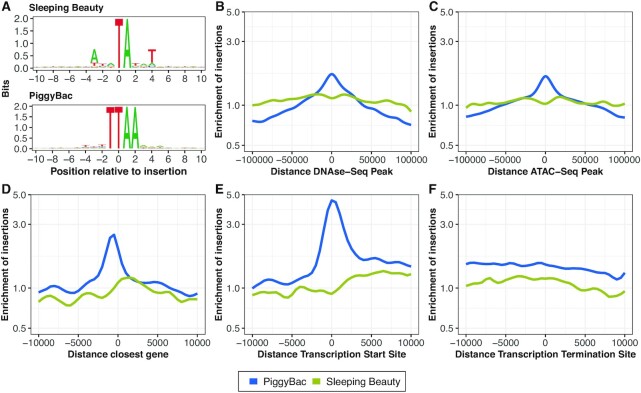
Genomic feature preferences of Sleeping Beauty and PiggyBac insertion sites. (**A**) Nucleotide preference (bits) around the insertion sites. (B–F) Fold enrichment of (**B**) insertion sites around DNAse-Seq peaks. (**C**) Insertion sites around ATAC-seq peaks. (**D**) Insertion sites relative to the closest gene body. Negative values represent loci upstream of genes. (**E**) Insertion sites around Transcription Start Sites. The transcription starts at position 0 with positive values denoting the transcribed region. (**F**) Insertion sites around Transcription Termination Sites. The transcription terminates at position 0 with negative values denoting the transcribed region. For panels B–F, enrichments were calculated as proportion ratios relative to random TA sites for Sleeping Beauty and random TTAA sites for PiggyBac in 500 bp genomic windows. Loci overlapping with the positions of the features were encoded as 0.

First, we found that both transposon systems display a significant bias regarding the local sequence context around their insertion sites (Figure 3A). This bias extends beyond the known consensus sequence of TA / TTAA. In line with previous research (35), the sequence bias appears particularly pronounced for SB.

Second, we found that SB and PB favour proximity to transcription start sites, transcription termination sites, and gene bodies (Figure 3D–F). Specifically, the mean absolute distance to the closest transcription start site (Figure 3E) was 97 kb among the PB insertion sites compared to 173 kb among the control sites (SB: 158 kb versus 171 kb; *p*-values for PB and SB < 10^−4^).

Third, we found that transposon insertion sites correlate with chromatin accessibility data (Figure 3B and C). Notably, PB strongly favours proximity to open chromatin regions identified by ATAC-seq (Figure 3C), with the mean absolute distance from PB insertion sites to the closest ATAC-seq peak (66 kb) being less than half of the equivalent distance for random TTAA sites (180 kb) (SB: 89 kb versus 188 kb, *p*-values for PB and SB < 10^−4^). Moreover, we found transposon insertions to be strongly associated with chromatin states in mESC (chromHMM states in mESC: 54,55) in accordance with previous research (38) (Supplementary Figure S1). Interestingly, the associations with both genic and chromatin-related features are much less pronounced among our SB insertions, supporting earlier findings that SB is relatively unbiased with regard to many genomic and epigenomic features (34–37). Note that we based the analysis of chromatin-related features on chromatin data generated in mESC due to their greater generalizability in the downstream analysis. A similar analysis based on B-cell data showed qualitatively no difference.

### Genomic and epigenomic features allow for accurate prediction of transposon insertion sites

Having found correlations between the aforementioned features and transposon insertion sites, we asked to what extent these can be used to model the background distribution of insertion sites. To this end, we trained a random forest classifier to distinguish between observed insertion sites (positives) and an equal number of TA/TTAA sites randomly sampled from the genome (negatives).

A potential caveat with training a mutagenesis model using data from mutagenesis screens is that insertion counts reflect both mutagenesis and selection. Our approach to limit the confounding effect of selection when training the mutagenesis model was two-fold: First, we considered unique insertion sites and discard coverage information, assuming that the read coverage of insertion site reflects clonal expansion (selection). Second, we used predictive features that a priori have a role in the mutagenesis (insertion) process but not in the selection process. Moreover, in the few datasets for which matching unselected samples were available, we assessed whether the mutagenesis model yielded similar predictions be trained on unselected samples or on selected samples.

First, we investigated the predictive power of the individual features in our DLBCL datasets, finding interesting differences between the SB and the PB datasets. For instance, we found that SB insertions (Supplementary Figure S4A) can be well predicted by the local sequence context (AUC = 0.87) but are poorly predictable from other features. In contrast, for PB insertions, the sequence context mattered less (AUC 0.70) than for SB insertions, whereas other features, including the distances to the closest DNAse-seq peak (AUC 0.57) and to the closest ATAC-seq peak (AUC 0.54), showed higher predictive power for PB than for SB (Supplementary Figure S2B). This is in line with our analysis of individual features and with previous studies (41).

To assess the generalizability of our models, we repeated this analysis in multiple previously described datasets (23,25,37,46) as well as the tail samples generated in our DLBCL experiments, which serve as negative, unselected controls. Overall, we found the predictive power of the various individual features to be similar between datasets (Supplementary Figure S2A and B), with some noticeable exceptions. Insertions in our DLBCL SB datasets are less predictable than insertions in other SB datasets for most of the features. Moreover, PB and SB insertion sites in two datasets generated in mESC without selective pressure (37) are systematically more predictable than the other datasets. For features based on data from mESC (e.g. DNAse, chromatin states), this is expected given that insertion data and feature data were generated in the same cell type. Also, the authors of this study acknowledge that the mESC insertion sites show a restriction site bias (21,37). Moreover, it has been suggested that differences in the insertion behaviour of transposon systems might be due to cell type-specific effects (41) or different transposase subtypes (37). These variations indicate that it might be relevant to train mutagenesis models specifically to each dataset.

**Figure 4.**
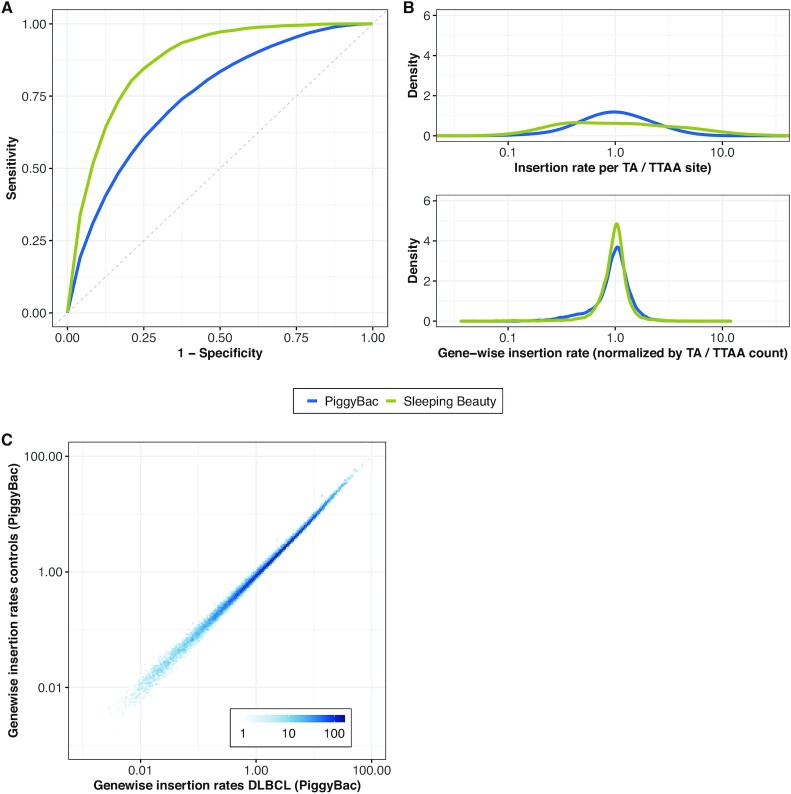
Characterization of the predictions obtained from our mutagenesis model in our DLBCL datasets. (**A**) ROC curves for the classification of observed insertion sites against an equal number of random TA sites (Sleeping Beauty) and TTAA sites (PiggyBac) by our mutagenesis model. ROC curves show the performance of our mutagenesis model on an unseen subset of the data (10%). (**B**) Density of the insertion rates for every TA and TTAA site in the genome (top) and aggregated for over every gene (bottom). Insertion rates are log-centered. (**C**) Genewise insertion rates obtained from the mutagenesis model trained on PiggyBac insertions using the DLBCL samples (x-axis) and the tail samples (unselected, y-axis) for training.

Even though the specificity of the unselected mESC dataset can be argued to explain the much different behaviour of the mutagenesis model, this raises the concern that the mutagenesis model trained on the selected data is confounded due to oncogenic selection. Therefore, we investigated the validity of the concern that the mutagenesis model learns a background distribution biased by oncogenic processes. To this end, we trained the mutagenesis model both on the DLBCL PB samples and on the corresponding tail samples and compared the gene-wise insertions rates. We found a high correlation (ρ = 0.99, *p*-value < 10^–4^, Figure 4C), suggesting that the mutagenesis model successfully avoids learning oncogenesis-related biases. This conjecture further strengthened the predictive power of the single features in the mutagenesis model being very similar in the tail samples and in the DLBCL samples for both transposon systems (Supplementary Figure S2).

Moreover, we found chromatin states to be highly predictive of transposon insertion sites, as previously suggested (38). However, we found stark differences in the predictive power of chromatin state data (chromHMM) obtained from different sources. For instance, contrary to expectations, chromatin states generated from B-cells (56) had much less predictive power than chromatin states generated from mESC (54,55) in our DLBCL datasets, which is a B-cell malignancy (AUC-SB = 0.5 versus 0.56, AUC-PB = 0.54 versus 0.65, Supplementary Figure S2). In addition, chromatin state data are not widely accessible for different cell types. For these reasons, we assessed whether chromatin state data could be omitted from our final joint model comprising all features. We compared the performances of the joint model with and without chromatin state data and found no substantial difference (Supplementary Figure S2), suggesting that chromatin-related effects can be sufficiently modelled using ATAC-seq and DNAse-seq data. Thus, we decided not to include chromatin state data in our final mutagenesis model.

Before integrating the joint mutagenesis model into Transmicron, we analysed its potential implications on cancer gene detection in more depth. First, we generated ROC curves for both DLBCL datasets, finding that the mutagenesis model predicts both SB insertions (AUC = 0.87) and PB insertions (AUC = 0.75) with high accuracy (Figure 4A). Second, we used the trained mutagenesis model to predict insertion rates, for every TA / TTAA site in the genome (Figure 4B and Equation 4). We find that predicted insertion rates of individual sites span up to two orders of magnitude for both SB and PB insertions, with insertion rates showing a larger spread for SB than for PB. This is in line with previous findings showing that SB demonstrates strong preferences regarding the local sequence context around TA sites (35). Importantly, insertion rates of individual SB sites varied as much as 60-fold between the lower and upper 5%-ile of the data with rates for PB sites showing only a 15-fold variation (Figure 4B). However, when averaged at the gene level $( {{\Lambda _j}} )$ and normalized by the number of TA / TTAA in each gene, this variation reduced down to 2-fold for SB and 4-fold for PB (Figure 4C). This averaging effect was more pronounced for SB than for PB, likely because SB insertions are mostly determined by the sequence context which is likely to vary across insertion sites within a gene more independently than the genomic and epigenomic context.

A major consequence of this averaging effect might be that the plain number of TTAA / TA sites remains a major determinant of expected insertion counts in a gene under neutral selection, potentially mitigating the need for a mutagenesis model.

### Mutagenesis background estimate increases precision in finding cancer genes

**Figure 5.**
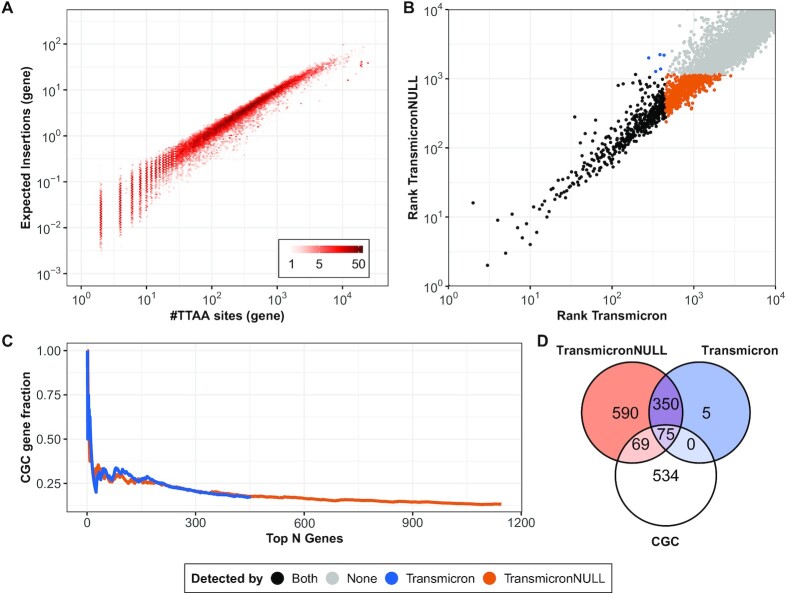
Effect of our mutagenesis model on cancer gene prediction in our DLBCL PiggyBac screen. (**A**) Relationship between the number of TTAA sites and the expected number of PiggyBac insertions in each gene derived from Transmicron (with mutagenesis correction). (**B**) Relationship between the ranks of each gene derived from Transmicron and TransmicronNULL (controlling only for the number of TTAA sites in each gene). Genes were ranked by *p*-value. If two or more genes had identical *p*-values, the genes were assigned the same rank using the highest value. Only the top 10 000 genes are displayed. (**C**) Comparison of the precision of Transmicron and TransmicronNULL. The proportion of CGC-genes among sets of top N genes detected by each method is shown. (**D**) Venn Diagram showing the overlap of the CGC with the genes detected by Transmicron and TransmicronNULL.

Subsequently, we explored the effects of adding the joint mutagenesis model to Transmicron. First, we generated expected insertion counts for every gene and analysed to what extent these expectations deviate from expectations controlling only for the number of TA/TTAA sites in each gene as in TransmicronNULL. We focused our analysis on PB insertions, where we found higher variation in gene-wise insertion rates than in SB. The number of TTAA sites and the expected number of PB insertions strongly correlated (ρ = 0.98, *p*-value < 10^−4^) (Figure 5A). This suggests that, while PB does favour certain TTAA sites and genes, the major driver of gene-wise insertion counts is the number of TTAA sites in each gene. Consistent with this observation, the gene ranking of TransmicronNULL and Transmicron agreed very well, especially among the low-ranking, i.e. high confidence, genes (Figure 5B). Among higher ranking genes, differences in the ranks obtained by both methods increased. Moreover, there was no systematic difference in CGC gene fraction across the set of top N genes sorted by *p*-value (Figure 5C and Supplementary Figure S3A). This supports the assumption that SB and PB transposon systems insert in a sufficiently unbiased way to be effective at identifying high-priority cancer driver genes without explicitly controlling for mutagenesis.

However, comparing the final sets of genes identified by Transmicron and TransmicronNULL (i.e. the end-points of the curves in Figure 5C and Supplementary Figure S3A and C), we observed that including the mutagenesis model reduced the number of detected genes and simultaneously improved precision. The mutagenesis model reduced the number of genes detected by 25% for SB (from 109 to 82) and 60% for PB (from 1,084 to 430), while increasing the CGC gene fraction from 26% to 31% for SB and from 13% to 17% for PB (Figure 5 C and D and Supplementary Figure S3A and B). These results suggest that the mutagenesis model help identify cancer genes by removing putative false positive CIS, in line with previous research on transposons insertion behaviour (20,39).

Next, we assessed the generalizability of these findings using four previously described datasets. For all datasets, the Transmicron with the mutagenesis model detected fewer candidate genes with a higher CGC gene fraction than TransmicronNULL. Second, the fraction of CGC genes for Transmicron was greater or equal than for any other methods in all datasets and across gene rank cut-offs (Supplementary Figure S3C).

As we found that the mutagenesis model of Transmicron cannot solely explain its strong performance relative to other methods, we investigated the impact of other conceptual differences between the methods. To assess the differences between gene-based (Transmicron, SB Driver) and window-based (PRIM, CIMPL) methods, we ran Transmicron based on non-overlapping genomic windows of 10 kb (Transmicron10kb), finding its precision to drop considerably (Supplementary Figure S3C). However, we found that Transmicron10kb still improves over PRIM and CIMPL with window size and scale parameters, respectively, set to 10 kb. This suggests that the improvements achieved by Transmicron are, at least in part, due to other elements of our modelling approach. For instance, TransmicronNULL, which differs from SB Driver only by the selection model, appeared to be more robust to low numbers of samples than SB Driver (Figure 2A, DLBCL SB 11 samples) which showed strong performance in datasets with >100 samples (Supplementary Figure S3C). Transmicron is more robust to low numbers of samples, possibly because it is based on the Poisson distribution instead of the chi-squared approximation. In addition, our GLM-based approach combines the inter-sample signal, as successfully introduced by SB Driver (27), with the intra-sample signal (insertion counts), potentially increasing sensitivity in datasets with low numbers of samples. As an additional robustness test, we validated the results from our DLBCL PB screen using a second catalogue of cancer genes, namely the DLBCL genes published in Reddy *et al.* (53) to compare the results of Transmicron to the results of PRIM and of CIMPL (Supplementary Figure S3D). Similar to the findings using the CGC, we found Transmicron to detect a higher proportion of cancer genes than CIMPL, PRIM and TransmicronNULL, suggesting the improvements are not driven by the choice of cancer gene catalogue.

To further demonstrate that Transmicron can correct for differences in the insertion site preferences of different transposon systems, examined whether Transmicron increases the similarity between results from SB and PB screens, relative to existing methods. We reasoned that statistical methods that correct for transposon system biases better than other methods should report more consistent gene lists when applied to two distinct transposon systems. Therefore, we examined whether Transmicron increases the gene list similarity between results from SB and PB screens, relative to existing methods. Specifically, we compared the Jaccard Index (Intersection over Union of two lists) of the genes detected by Transmicron in our two DLBCL screens (SB and PB) to the Jaccard Index found using CIMPL (14) and PRIM (20) (Supplementary Figure S3E). The Jaccard Index calculated using Transmicron (0.09) is at least twice as high as the Jaccard Index among existing methods (PRIM, 0.04, CIMPL, 0.02). This indicates that our mutagenesis model successfully controls for transposon type-specific effects.

### Transmicron results are robust against the specific choice of training data

**Figure 6.**
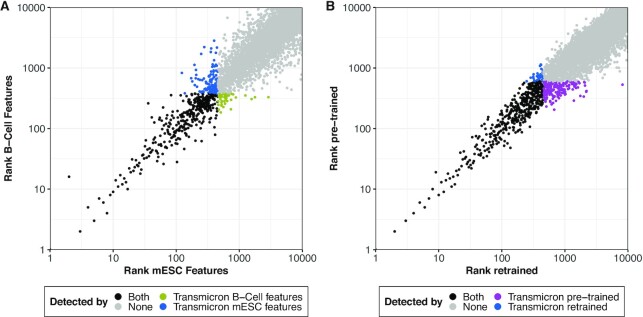
Comparison of Transmicron using different training data. Gene ranks obtained training the mutagenesis model of Transmicron on different data, testing on our DLBCL PiggyBac screen. Genes were ranked by p-value. If two or more genes have identical p-values, the genes were assigned the same rank using the highest value. Only the top 10,000 genes are displayed. In both panels, the Transmicron version displayed in blue is identical to the version used in the previous analyses. (**A**) Relationship between the ranks of each gene derived from Transmicron trained with chromatin accessibility data from B-Cells and from mESC. (**B**) Relationship between the ranks of each gene derived from Transmicron re-trained directly on the DLBCL screen and Transmicron pre-trained on a dataset of unselected insertions in mESC. Here, the same set of chromatin accessibility data from mESC was used in both versions of Transmicron.

The mutagenesis model of Transmicron is, in part, based on cell-type-specific data. The results presented so far were generated by training the mutagenesis model with DNA accessibility data from mESC. As we have seen in our analysis of individual features, mESC-based ATAC-seq and DNAse-seq data constitute reasonable surrogates for the chromatin accessibility in our DLBCL dataset. To investigate to what extent the results of Transmicron are affected by the choice of cell-type-specific DNA accessibility data, we replaced the mESC DNA accessibility data with B-cell-specific DNA accessibility data and examined the impact on our result (Figure 6A).

First, we observed the ranks of genes identified by both versions of the background model correlate very strongly. Second, we observed interesting differences between the two versions of the background model regarding the ability of Transmicron to remove putative false positive CIS. We found that using the more appropriate B-cell features removed a considerable number of genes that had been significant when mESC features were used while adding few extra genes. Notably, the genes impacted by the differences in cell type tend to be highly ranked genes, suggesting that controlling for cell type-specific features does not affect the high-confidence candidate genes identified by Transmicron. This is perhaps unsurprising, given that the mutagenesis model showed only limited impact on the ranking of the high confidence genes, even when compared to TransmicronNULL.

Since we found Transmicron results to be robust against the choice of DNA accessibility data, we intended to provide a pre-trained version, enabling the end-user to implement Transmicron without the need to acquire dedicated DNA accessibility data and retrain the computationally intensive mutagenesis model. To this end, we compiled Transmicron with a mutagenesis model that was trained on insertion data generated in mESC without selective carcinogenic pressure (Transmicron pre-trained) and compared it to a model that was trained on insertion data from our DLBCL PB data (Transmicron retrained). We found that the pre-trained and retrained results show a high degree of correlation, with differences only in the highly ranked genes (Figure 6B). Likewise, the expected insertion counts for each gene generated by the two versions of Transmicron correlate very strongly (ρ = 0.99, *p*-value < 10^−4^, Supplementary Figure S4).

Overall, this analysis suggests that Transmicron can take advantage of retraining on specialized experimental data while not affecting the detection of high-priority cancer driver genes.

## DISCUSSION

Identifying CISs from transposon mutagenesis screens is an important task in the quest to understand the molecular dynamics of oncogenesis. Previous studies suggested that CIS detection suffers from biases arising from benign transposon insertion site biases (20,29,39). Thus far, this problem has been addressed by comparing insertions from tumour samples with insertions from control samples which were subjected to minimal selective pressure (20). As generating control samples is labour-intensive, an in-silico correction for insertion biases may be preferable. However, existing computational tools used to analyse transposon screens do not sufficiently integrate knowledge on insertion site preferences, leading to a considerable number of putative false positive CISs (39). Here, we introduced Transmicron, the first computational method to prioritize candidate cancer driver genes from transposon mutagenesis screens while systematically correcting for multiple insertion site biases.

We started by establishing TransmicronNULL, a GLM-based tool to model oncogenic selection and showed that it can accurately identify cancer driver genes independently of the correction for insertion site biases. In all datasets we tested, TransmicronNULL achieved a precision that was at least on par with the best existing tool in each dataset. This illustrates the power of our selection model, which combines well-established Poisson statistics as applied by Bergemann *et al.* (21) (PRIM) and Sarver *et al.* (22) (TAPDANCE), with GLM-based testing for gene-wise selection coefficients while correcting for variations in per sample sequencing depth. Therefore, Transmicron is the first method to combine information on both the distribution of insertions across samples and on the total number of unique insertions across genomic regions. Previous methods have either summed insertion counts across samples (18,21,22,24), thereby disregarding information on the distribution of insertion across samples, or they have considered only the number of samples with insertions in a particular gene (27), losing intra-sample information. Our findings suggest that this modelling choice matters in particular in experiments with a low number of samples.

We then investigated to what extent cancer gene identification is affected by mutagenesis correction, finding that the effects of mutagenesis correction are in line with theoretical expectations: While transposon insertion biases implied a considerable variation of insertion probabilities between single sites, these variations reduced considerably when averaged along the length of entire genes. Eventually, the number of potential insertion sites appeared to be the main factor determining the neutral insertion counts in a gene. Consequently, gene rankings were not strongly affected by mutagenesis correction, particularly among the top-ranking genes. This supports the assumption behind many mutagenesis screens that SB and PB can identify high-priority cancer driver genes without explicit mutagenesis controls.

However, the mutagenesis model had a notable effect on the significance cut-off, whereby mutagenesis correction considerably reduced the number of genes detected as cancer genes given the same FWER threshold. The mutagenesis model, which is trained on both intragenic and intergenic regions, learns that PB and SB favour genes and other open chromatin regions as target sites under non-selective conditions. Thus, the expected insertion counts among all genes are, on average, higher when mutagenesis correction is included than when only gene length is controlled for. This increase in expected insertion counts causes some genes to no longer be detected as significant when mutagenesis correction is applied. This adjustment of the statistical threshold is beneficial as it increases the proportion of known cancer genes among the detected genes. This can have implications for researchers to ‘know when to stop’, when looking at entire candidate lists of a screen.

Having examined the effect of our mutagenesis correction, we investigated how mutagenesis correction using Transmicron is best implemented in practice. First, we demonstrated that one feasible approach is to train the mutagenesis model directly on the experimental data at hand by showing this does not lead to major cancer-related biases in the background distribution. This might be due to the features incorporated in the mutagenesis model not being strongly related to oncogenic processes. For instance, it is unlikely that the sequence context of carcinogenic insertions differs significantly from non-carcinogenic insertions. A second reason might be that the mutagenesis model considers unique insertions, disregarding coverage information. Thus, the ratio of carcinogenic insertions to non-carcinogenic insertions might be small, implying that the mutagenesis model learns from data with a majority of unselected insertions. This may cause the bias from oncogenic activity to be insignificant.

Second, we investigated the feasibility of using an ‘out-of-the-box’, pre-trained version of the mutagenesis model. We found that training the mutagenesis model on unselected insertions in mESC (rather than on the mutagenesis screen itself), has a significant effect only on lower-priority candidate cancer genes. Similarly, we found cell-type-specific mutagenesis features to have limited impact on the high-priority cancer genes in our data. This suggests that researchers primarily interested in high-priority candidate cancer genes may as well use the pre-trained version of the mutagenesis model. Researchers investigating genes that are less obviously implicated in oncogenesis may benefit from retraining Transmicron on their experimental data or use cell-type specific features to control for.

Transmicron is publicly available as a flexible tool accommodating different user requirements. We provide a precalculated background distribution based on unselected mESC insertions which we expect to be sufficient for most users. Moreover, Transmicron enables users to retrain the mutagenesis model using our predefined features, or, alternatively, supplying any BED-formatted feature they wish to control for. This option to supply user-defined features is aimed at use cases for which cell-type specificity is more important than for our DLBCL screens. For users interested only in the selection model, we offer the option of running TransmicronNULL correcting only for the distribution of TA/TTAA sites. Moreover, while previous tools call CISs either on genes or genomic windows, users can choose to run Transmicron on genes, genomic windows of any size, or supply their own GTF-formatted annotation (e.g. promoters, enhancers). Finally, Transmicron can be run on both SB and PB without modification. One limitation of our current model is that we excluded the donor chromosome, following previous approaches (27): Transmicron cannot accurately model the tendency of transposons to insert close to their donor locus (′local-hopping′) (9). Notably, not all regions on the donor chromosome are affected by local-hopping to the same degree, i.e. regions far away from the donor locus are less strongly affected. The chromosome factors introduced in Equations (4) and (5) cannot capture variations within chromosomes. Future research could investigate how local-hoping could be modelled, e.g. by learning the effect of the distance to the donor site. A difficulty here is that the number of established oncogenes in a single chromosome is much smaller than genome-wide and therefore statistical significance is hard to reach.

In sum, Transmicron provides a valuable tool for cancer gene detection from mutagenesis screens. The selection model behind Transmicron makes conceptual advances by explicitly considering both the inter-sample as well as the intra-sample distribution of insertions. The mutagenesis model offers a way to systematically analyse the impact of benign insertion biases of transposon systems and Transmicron is the first method to demonstrate how these biases can be controlled in a statistically robust way.

## DATA AVAILABILITY

Transmicron is freely available as an open-source software tool in the GitHub repository (https://github.com/gagneurlab/transmicron). A permanent DOI pointing to the exact version used for this manuscript is provided at: https://doi.org/10.5281/zenodo.7388676. Sequencing data have been deposited at GEO (accession number: GSE214379).

## Supplementary Material

gkac1215_Supplemental_File
