# Supplementary material for: Transmicron: accurate prediction of insertion probabilities improves detection of cancer driver genes from transposon mutagenesis screens
Source: Nucleic Acids Res. 2023 Jan 9;51(4):e21. doi: 10.1093/nar/gkac1215 (PMC9976929; doi:10.1093/nar/gkac1215)
Supplement: gkac1215_Supplemental_File [file gkac1215_supplemental_file.pdf]

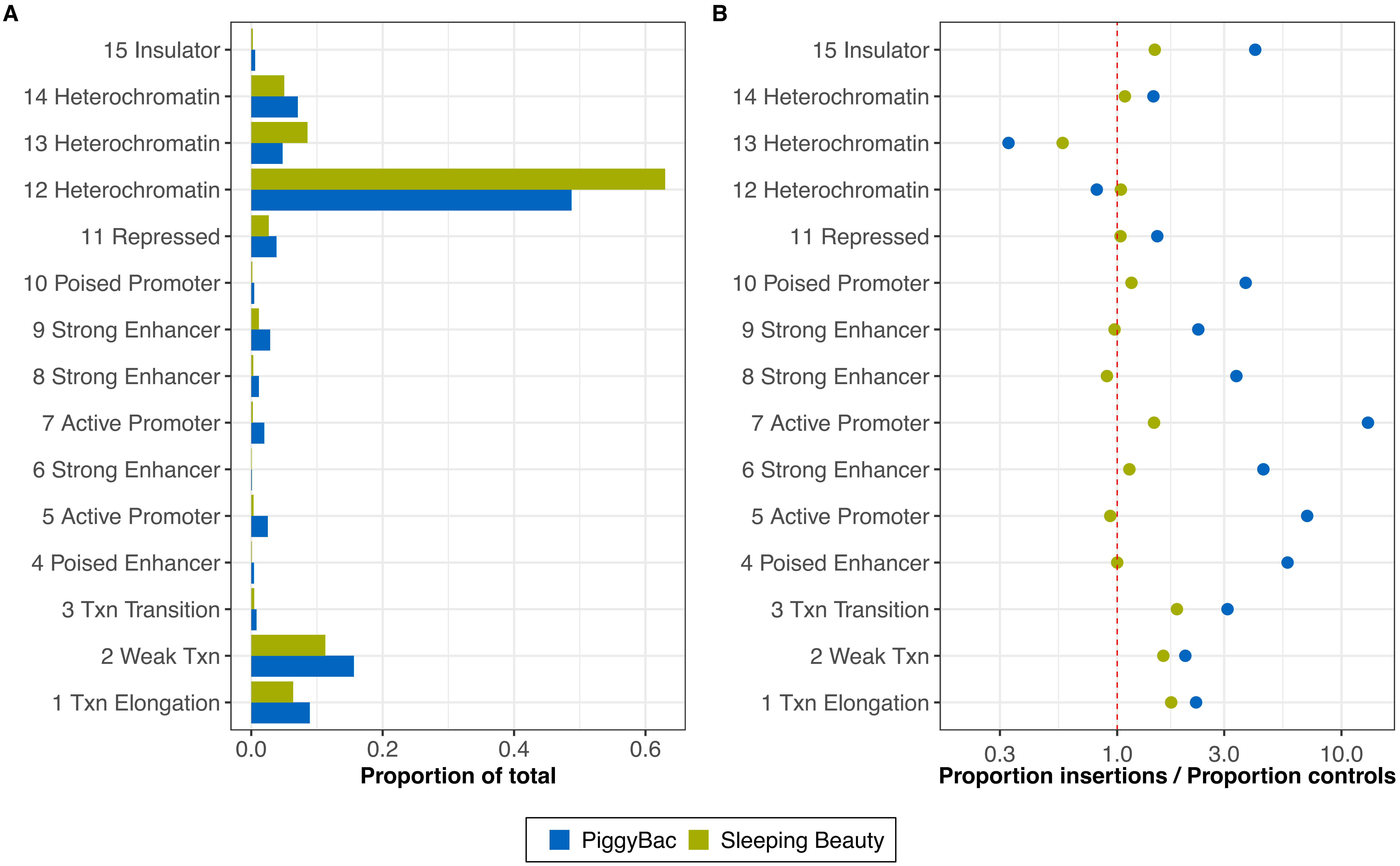

Figure S1: Chromatin states (54, 55) associated with insertion sites. **A)** Share of the whole genome per chromatin state. **B)** Odds ratios of insertion sites relative to random controls for each chromatin state. Values larger than one indicate enrichment of insertion sites relative to random controls.

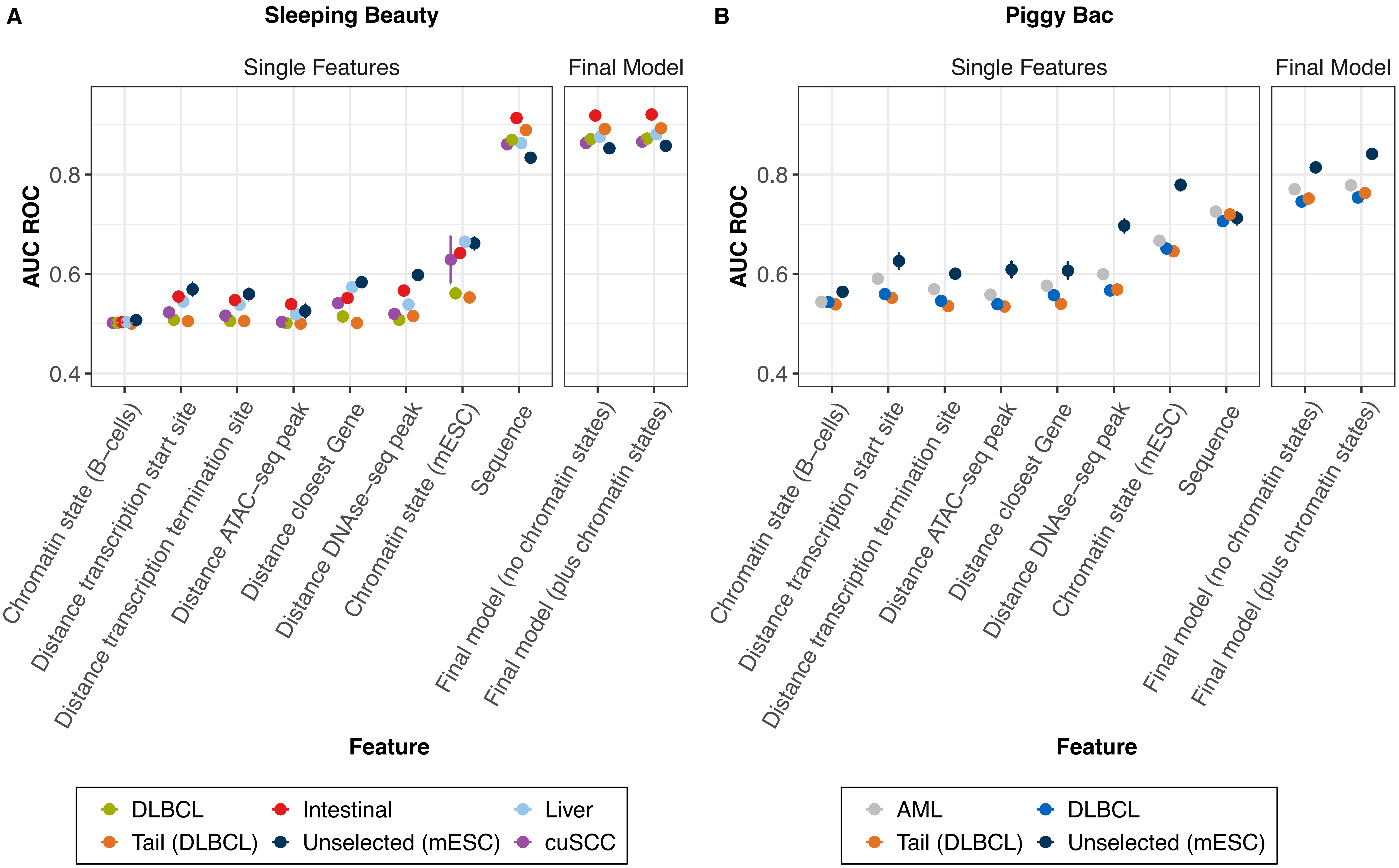

Figure S2: Predictive performance of our mutagenesis model based on different features in various datasets. The area under the ROC curves is displayed for the classification of observed insertion sites and an equal number of random TA sites (Sleeping Beauty, **Panel A**) or TTAA sites (PiggyBac, **Panel B**). ROC curves were calculated for Random Forest classifiers that were trained based on different features in various transposon screens. The models were tested on an unseen subset of each dataset (10%). The final model was trained on the combined single features. When chromatin states were added to the final model, chromatin states from insertions in mESC were used.

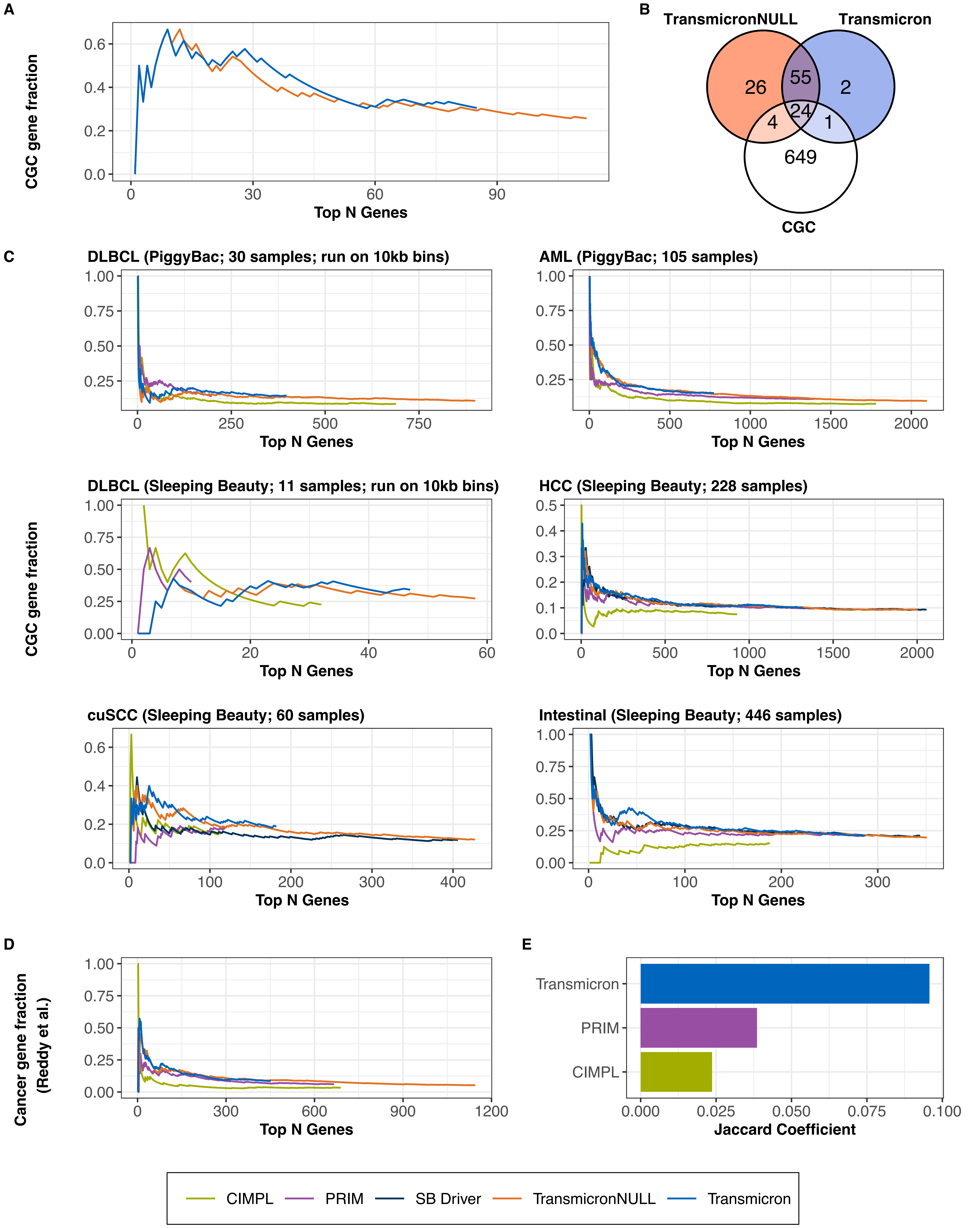

Figure S3: Effect of the mutagenesis correction introduced by Transmicron. **A)** Comparison of the precision of Transmicron and TransmicronNULL in our DLBCL Sleeping Beauty screen. The fraction of CGC-genes among sets of top N genes detected by each method is shown. **B)** Venn Diagram showing the overlap of the CGC with the genes detected by Transmicron and TransmicronNULL in our DLBCL Sleeping Beauty screen. **C)** Comparison of the precision of Transmicron to existing tools as well as to TransmicronNULL. The fraction of CGC-genes among sets of top N genes detected by each method is shown. Results from Transmicron, TransmicronNULL, PRIM, and SB Driver Analysis were sorted by gene-wise p-value. If for any method, a gene is detected multiple times, the lowest p-value is used. Results for CIMPL were sorted by its peak height metric because this has yielded higher precision than sorting by p-values. If two or more genes have identical p-values, the genes were assigned the same rank using the highest value. Curves stop at the last significant gene according to the respective method. Unless stated otherwise, Transmicron and TransmicronNULL were implemented on genes. **D)** Comparison of the precision of Transmicron to existing tools as well as to TransmicronNULL in our DLBCL PiggyBac screen. The fraction of DLBCL genes also indexed in Reddy et al. (53) among sets of top N genes detected by each method is shown. Results from Transmicron, TransmicronNULL, and PRIM were sorted by gene-wise p-value. If for any method, a gene is detected multiple times, the lowest p-value is used. Results for CIMPL were sorted by its peak height metric because this has yielded higher precision than sorting by p-values. If two or more genes have identical p-values, the genes were assigned the same rank using the highest value. Curves stop at the last significant gene according to the respective method. Transmicron and TransmicronNULL were implemented on genes. **E)** Bar chart comparing the similarity of the results from our DLBCL Sleeping Beauty and DLBCL PiggyBac datasets. The Jaccard Coefficients between the lists of genes detected in the Sleeping Beauty and the PiggyBac screen using Transmicron, PRIM, and CIMPL are shown.

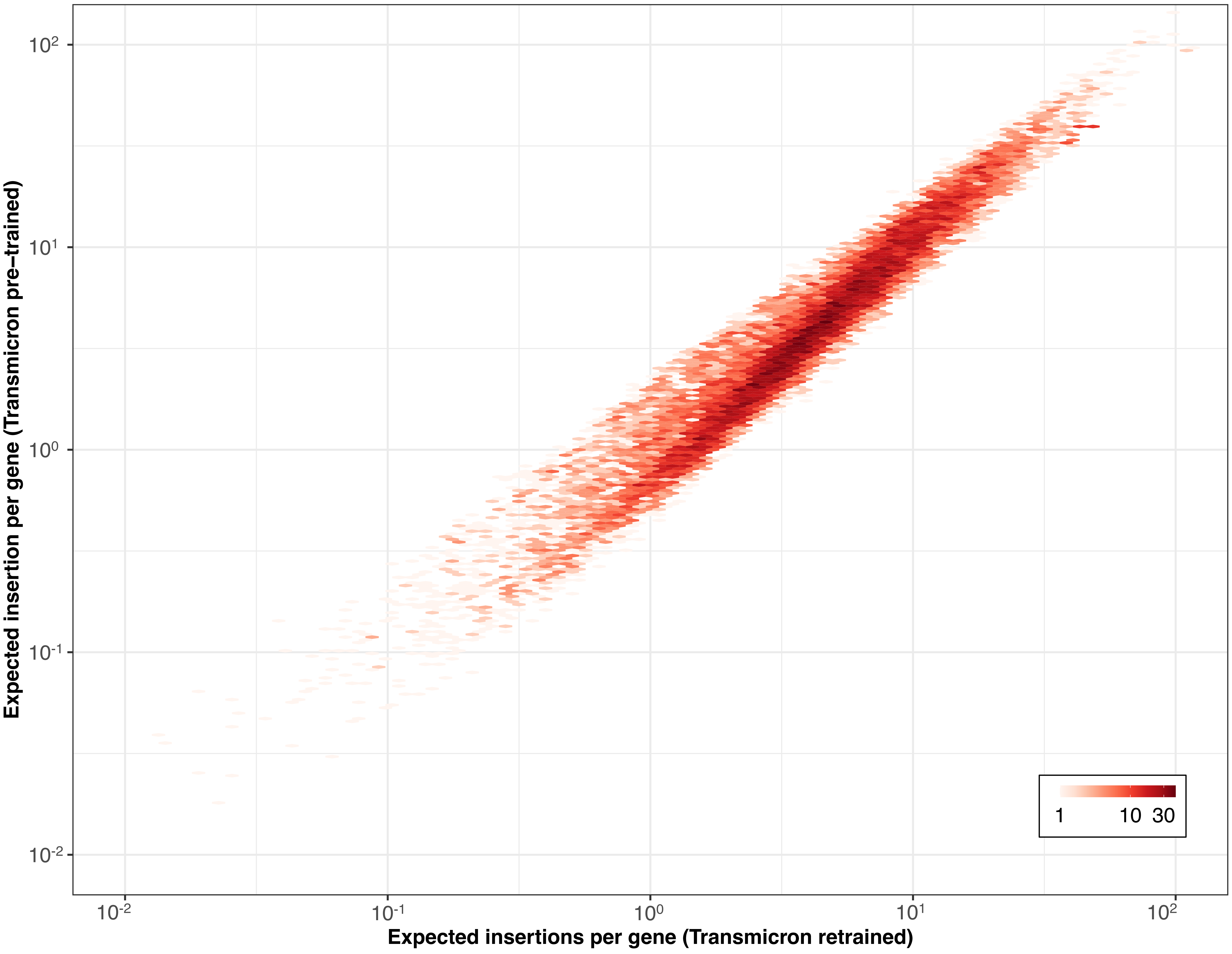

Figure S4: Relationship between the expected number of PiggyBac insertions in each gene for two versions of Transmicron. Expectations were obtained from Transmicron either trained directly on our DLBCL screen (retrained), or from Transmicron trained on a dataset of unselected insertions in mESC (pre-trained).
